# Chiroptical Performances in Self-Assembled Hierarchical Nanosegregated Chiral Intermediate Phases Composed of Two Different Achiral Bent-Core Molecules

**DOI:** 10.3390/ijms232314629

**Published:** 2022-11-23

**Authors:** Jae-Jin Lee, Sangsub Kim, Hiroya Nishikawa, Yoichi Takanishi, Hiroshi Iwayama, Changsoon Kim, Suk-Won Choi, Fumito Araoka

**Affiliations:** 1 Department of Advanced Materials Engineering for Information and Electronics, Integrated Education Institute for Frontier Science & Technology (BK21Four), Kyung Hee University, Yongin 17104, Republic of Korea; 2 Graduate School of Convergence Science and Technology, and Inter-University Semiconductor Research Center, Seoul National University, Seoul 08826, Republic of Korea; 3 Physicochemical Soft Matter Research Team, RIKEN Center for Emergent Matter Science (CEMS), Wako 351-0198, Japan; 4 Department of Physics, Kyoto University, Kyoto 606-8502, Japan; 5 UVSOR Synchrotron Facility, Institute for Molecular Science, Okazaki 444-8585, Japan

**Keywords:** self-assembly, chirality, bent-core molecules, circularly polarized luminescence, circular dichroism, helical nanofilaments

## Abstract

In this paper, chiral intermediate phases composed of two achiral molecules are fabricated by utilizing nanophase separation and molecular hierarchical self-organization. An achiral bent-core guest molecule, exhibiting a calamitic nematic and a dark conglomerate phase according to the temperature, is mixed with another achiral bent-core host molecule possessing a helical nanofilament to separate the phases between them. Two nanosegregated phases are identified, and considerable chiroptical changes, such as circular dichroism and circularly polarized luminescence, are detected at the transition temperatures between the different nanophase-separated states. The nanosegregated chiral phase—wherein the helical nanofilament and dark conglomerate phases are phase-separated—exhibits the highest chiroptical intensities. The luminescence dissymmetry factor, |g_lum_|, in this phase is amplified by an order of magnitude compared with that of another nanosegregated phase, wherein the helical nanofilament and nematic phases are phase-separated.

## 1. Introduction

**Figure 1:**
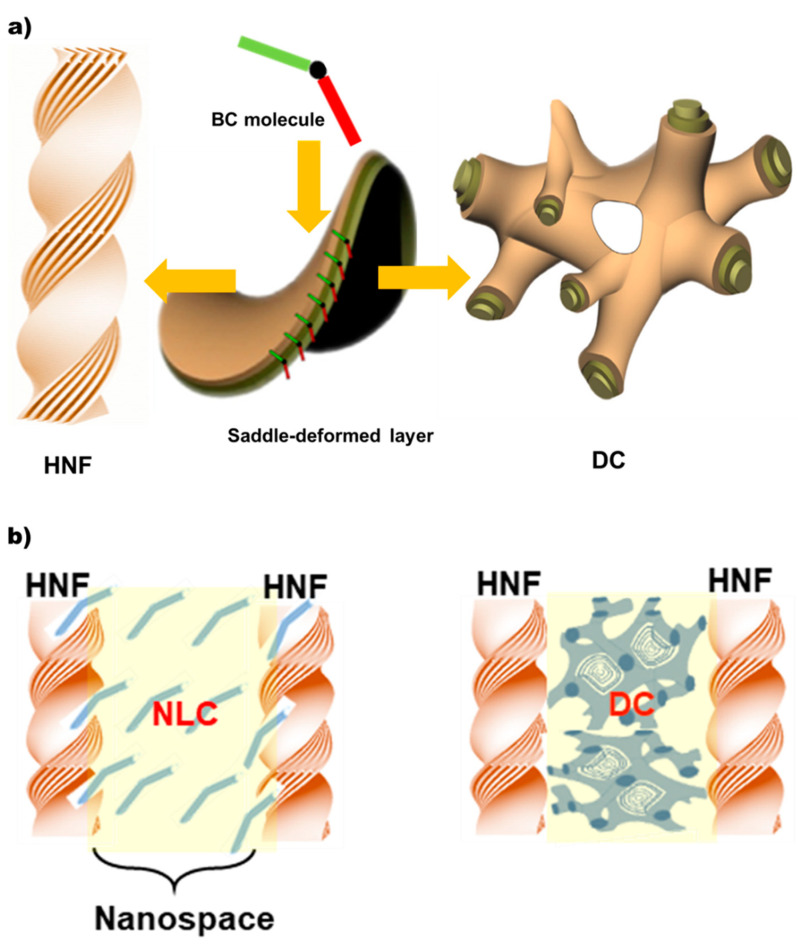
(**a**) Schematic representations of HNF and DC structures. The upper and lower halves of the BC molecule organize orthogonally, forming a saddle-deformed layer structure. The saddles assemble either the HNF or DC structures. (**b**) Two nanosegregated phases exhibit different hierarchical molecularly organized BC guest molecules embedded in the nanospaces generated between BC-host molecular HNFs. Illustrations on the left and right indicate <HNF/N> and <HNF/DC>, respectively. <HNF/N (or DC)> refers to a nanosegregated phase in which BC-1 and BC-2 are in the HNF and NLC (or DC) phases, respectively.

Chirality is a fascinating scientific topic because it is a universal phenomenon in nature [1]. Chirality-related topics have attracted interest in the field of liquid-crystal (LC) science [2,3,4,5], and indeed, some important properties in LCs, such as ferroelectricity, are induced through chirality [6]. Herein, we explore an interesting chirality-related phenomenon in nano-separated intermediate phases composed of two discrete achiral bent-core (BC) molecules. Achiral BC molecules [7,8] are well-known for spontaneous chiral resolution at the macroscopic scale despite the chiral-carbon-atom-free (hence, achiral) molecules. The so-called B4 phase [9,10]—often the lowest temperature phase of BCs—is one such chiral mesophase in which a helical nanofilament (HNF) superstructure is spontaneously self-assembled, as depicted in Figure 1a. In this case, structural chirality, observed as helical handedness, originates from the layered chirality of tilted smectic layers twisting along the HNFs. One highly similar type of chiral phase formed by BC molecules is the dark conglomerate (DC) phase [10,11] (Figure 1a). The DC exhibits optically resolved domains with strong optical activity, such as the B4 phase, but it appears to be more isotropic. The fundamental morphology of DCs comprises disordered focal conical structures, such as a lyotropic sponge structure, wherein the empty volume is filled with tilted smectic layers [10,11]. Although sponge-like structures do not have morphological chirality, it is understood that the observed macroscopic optical activity is attributed to the layered chirality of the tilted smectic layers, similar to the B4 phase.

Recently, circularly polarized luminescence (CPL) has been successfully induced in a nanosegregated intermediate phase in a mixed system consisting of a BC molecule (host) and a calamitic nematic LC (NLC) molecule (guest) blended with a luminescent dye [12]. By combining BC and calamitic molecules, nanophase segregation is initiated between the HNF and NLC [13,14,15,16,17]. In this phase-segregated nano/mesoscopic state, the structural chirality of the BC-molecule-originated HNF affects the NLC confined between the HNFs, forming an HNF-chirality-associated chiral superstructure, which is strongly supported by the amplified induced circular dichroism (CD) signal in the mixed-NLC molecular absorption band [14]. If luminescent dyes are doped in an NLC-organized chiral superstructure between BC-molecule-originated HNFs, these luminescent dyes co-assemble and obey the spatial order of the confined nanochiral structure of the NLC. Consequently, the luminescent dyes embedded therein exhibit CPL [12]. For this system fabricated using the NLC confined within the nanospace between the HNFs, the evaluated CPL performance is comparable with previously reported values for low-molecular-weight, chiral, organic compounds in solution [12]. However, developing a strategy to further amplify the CPL performance is vital for applying this system to potential applications in display [18], sensing [19], communication [20], security [21], and recording technologies [22].

In this study, we fabricated two intermediate phases (as illustrated in Figure 1b) using nanophase segregation between two BC molecules and hierarchical molecular self-organization. Furthermore, the dependence of their chiroptical performances on different hierarchical molecular organizations in two nanosegregated phases was investigated. In the temperature range of the B4 (HNF) phase of the host BC molecule, the guest BC molecule transited from an isotropic (Iso) to a crystalline phase through two mesophases, NLC and DC. We used polarized optical microscopy (POM) and X-ray diffraction (XRD) to identify the two meaningful nanosegregated phases in the binary BC mixture system. Next, we investigated the CD dependence on thermotropic polymorphism (NLC and DC) in the guest BC molecule confined in the nanospaces between the HNFs formed by the host BC molecule. Subsequently, CPL was investigated in these nanosegregated phases in which the guest BC was doped with a luminescent dye. CD and CPL were found to be strongly dependent on the thermotropic polymorphism (hierarchical molecular organization) of the BC guest molecules embedded in the BC-host molecular HNF-generated nanospaces. Interestingly, the luminescence dissymmetry factor, |g_lum_|, of the nanosegregated state of the HNF and DC was one order of magnitude higher than that of the nanosegregated state of the HNF and NLC.

## 2. Results

**Figure 2:**
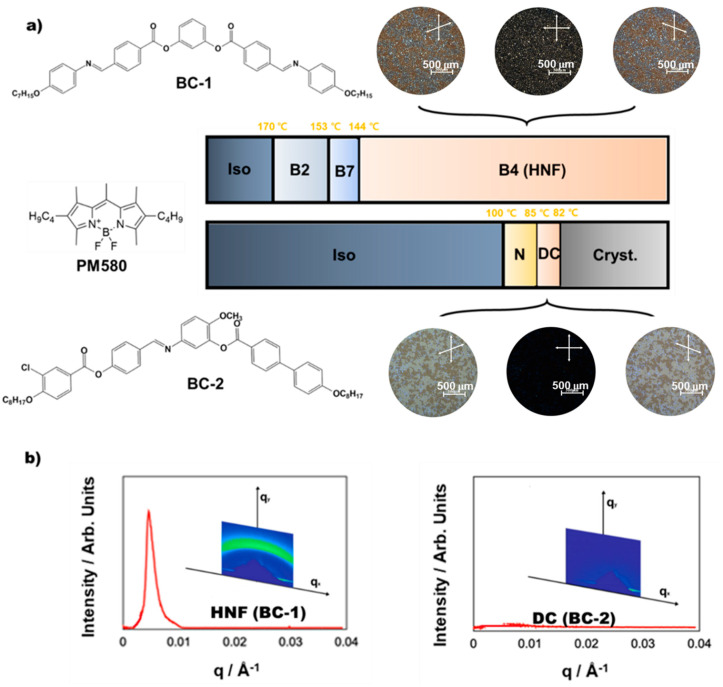
(**a**) Molecular structures, phase sequences (as determined during cooling), and typical POM images of two enantiomeric domains in the B4 (HNF) phase (BC-1) and DC phase (BC-2). The molecular structure of the PM580 luminescent dye is also depicted. (**b**) Carbon K-edge 2D RSoXS patterns of HNF (BC-1) and DC (BC-2) and their 1D profiles plotted on *q*. The triangular object in the 2D pattern is a projection of the dumper blocking the direct beam.

A mixture composed of BC-1 (host) and 40 wt.% BC-2 (guest) was prepared (M1 mixture). The chemical structures and phase sequences (as determined during cooling) of BC-1 and BC-2 are shown in Figure 2a. BC-1 only exhibited unique “Banana” mesophases found in typical BC molecular systems, such as the B2, B7, and B4 phases, consistent with previous studies, the structural foundation of which are polar-tilted smectic layers [23]. Conversely, BC-2 exhibited a calamitic N phase despite the BC molecule because the bend angle of BC-2 is slightly larger than that of the classic BC molecule. Furthermore, the BC-2 molecule transits to DC through the N phase. Two enantiomeric domains consisting of right- or left-twisted smectic-layer nanofilaments appeared under the de-crossed condition in BC-1 (in the B4 (HNF) phase), as shown in Figure 2a. Similarly, two enantiomeric domains formed by the layered chirality of the two different tilted smectic layers were also observed in BC-2 (in the DC phase). Although these chiral phases are very similar to each other, the texture of the DC phase of BC-2 is rather smooth and isotropic, whereas the chiral domains of the B4 phase of BC-1 are slightly birefringent. Because the HNF and DC phases are similar to each other, the DC phase in BC-2 has been mistakenly reported as an HNF phase [24,25]. The carbon K-edge resonant soft X-ray scattering (RSoXS) patterns of the HNF (BC-1) and DC (BC-2) are compared in Figure 2b, which clearly demonstrates the morphological differences between these states. RSoXS, whose energy corresponds to carbon K-edge absorption, is useful to analyze nanoscopic structures with a scarce electron density modulation, particularly in such morphological systems. The typical RSoXS pattern of the B4 HNF phase (65 °C) in the pure BC-1 shows a relatively sharp peak at the scattering angle corresponding to the half-pitch periodicity of the helical filaments (d ≈ 128.2 nm (q ≈ 0.005 × 10^−3^ Å^−1^)). Contrary, the typical RSoXS pattern of the DC (84 °C) of the pure BC-2 only shows featureless background scattering, unlike the HNF. This implies that, in the DC, there are no clear periodic structures in the scattering range with a pitch detectable with the present setup (d ≈ 18–250 nm).

**Figure 3:**
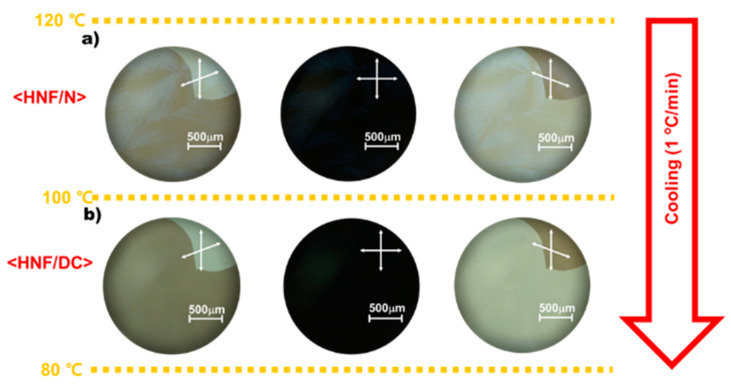
Typical POM images of (**a**) <HNF/N> and (**b**) <HNF/DC> appearing in M1 cooled at 1 °C min^−1^. The arrows indicate the polarizer orientations.

Figure 3 shows typical POM images of two notable nanosegregated phases appearing in M1 cooled at 1 °C min^−1^ from the Iso phase. Hereafter, to simply depict the nanosegregated phases in the mixed systems composed of the BC-1 host and the BC-2 guest, we employ the notation <phase of BC-1/phase of BC-2> [26]. During cooling, <HNF/N> appears—as shown in Figure 3a—in which dark images are preserved when the polarizers are crossed. By contrast, when the upper polarizer is slightly rotated clockwise (or counter-clockwise), two differently colored spherulite-containing domains appear; when the polarizer is rotated in opposite directions, the colors are exchanged. <HNF/DC> appears when M1 is cooled further, leading to the disappearance of spherulites, whereas the boundary between the two domains appears even clearer when the polarizers are de-crossed (Figure 3b). Note that the CD intensities of these domains are stronger, as mentioned later, and, thus, it is natural that the domain boundaries are visualized more clearly if they originate from the larger optical rotatory power. The temperature ranges of the two intermediate phases and the phase-transition temperature between the two phases measured based on POM are also summarized in Figure 3. Both differently colored domains appear when the polarizers are de-crossed, indicating that both chiral domains consist of two opposite chiral superstructures exhibiting opposite optical rotatory powers because the mixed system consists of only chiral-carbon-atom-free (hence, achiral) molecules. When chiral superstructures are spontaneously assembled by achiral molecules, two chiral superstructures exhibiting opposite chirality are generated with equal probability.

**Figure 4:**
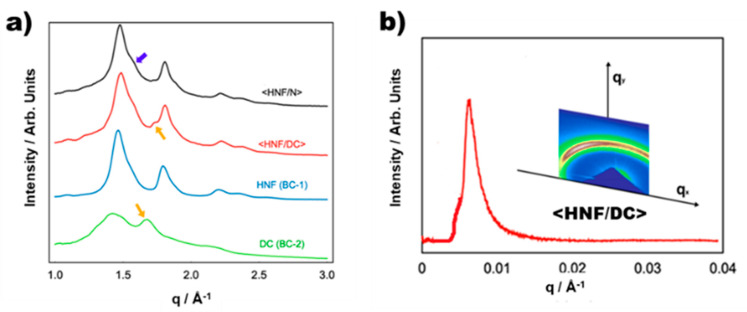
(**a**) Typical WAXD profiles generated in different temperature ranges for <HNF/N>, <HNF/DC>, pure HNF formed by BC-1, and pure DC formed by BC-2. (**b**) The 2D RSoXS pattern of <HNF/DC> at 90 °C and its 1D profile plotted on *q*. The triangular object in the leftmost part of the pattern is the dumper blocking the direct beam.

Figure 4a shows the typical wide-angle X-ray diffraction (WAXD) profiles generated in different temperature ranges for <HNF/N>, <HNF/DC>, pure HNF formed by BC-1, and pure DC formed by BC-2. Multiple diffuse peaks in the WAXD profile of the HNF (BC-1) are attributed to the intra-layer hexatic positional order, which is a typical feature of HNF phases [9]. The WAXD profile of the DC (BC-2) has fewer peaks with a broader shape and a lower intensity than that of the typical HNF. Although DCs in many BC materials show featureless WAXD profiles, the present type of WAXD profile has also been observed in some other BC molecules [27].

The WAXD profile of <HNF/N> is almost identical to that of the HNF, except for an ambiguous shoulder (indicated by a blue arrow), which appears to be added to the background. The added diffused and ambiguous shoulder may be attributed to an in-plane order in N (BC-2). Because the WAXD profile is a simple superposition of those for the pure HNF (BC-1) and N (BC-2), it is elucidated that <HNF/N> is a nano-separated state, wherein BC-1 and BC-2 are the HNF and NLC, respectively.

The profile of <HNF/DC> is also considered a superposition of the WAXD profiles of the pure HNF (BC-1) and DC (BC-2), as a weak but distinct peak (indicated by yellow arrows) originating from the DC (BC-2) is superimposed in the WAXD profile of the HNF (BC-1). Recently, it was reported that the nanoconfined DC phase within nanochannels forms the HNF structure instead of the DC structure [28]. The 2D RSoXS pattern and 1D profile of <HNF/DC> are presented in Figure 4b. For <HNF/DC>, at 90 °C, only one type of sharp signal is observed at *q* ≈ 0.0062 Å^−1^ (101.3 nm) similar to that observed for the HNF (BC-1), as shown in Figure 2b. This demonstrates the absence of other types of HNF structures converted from DC (BC-2) confined within the nanospaces. It is strongly suggested that <HNF/DC> is a nanosegregated phase, wherein BC-1 and BC-2 are in the HNF and DC phases, respectively, based on WAXD and RSoXS data.

These two notable nanosegregated phases, <HNF/N> and <HNF/DC>, were also identified in this mixture using differential scanning calorimetry (DSC), as shown in Figure S1a. Based on the POM, WAXD, and DSC observations, the phase diagrams of the binary mixture systems with different fractions are depicted in Figure S1b. Although three intermediate phases, <HNF/Iso>, <HNF/N>, and <HNF/DC>, are observed in this binary mixture system between the Iso and crystalline phases, we focused on two nanosegregated phases, <HNF/N> and <HNF/DC>, because significant chiroptical performances are expected in these phases.

**Figure 5:**
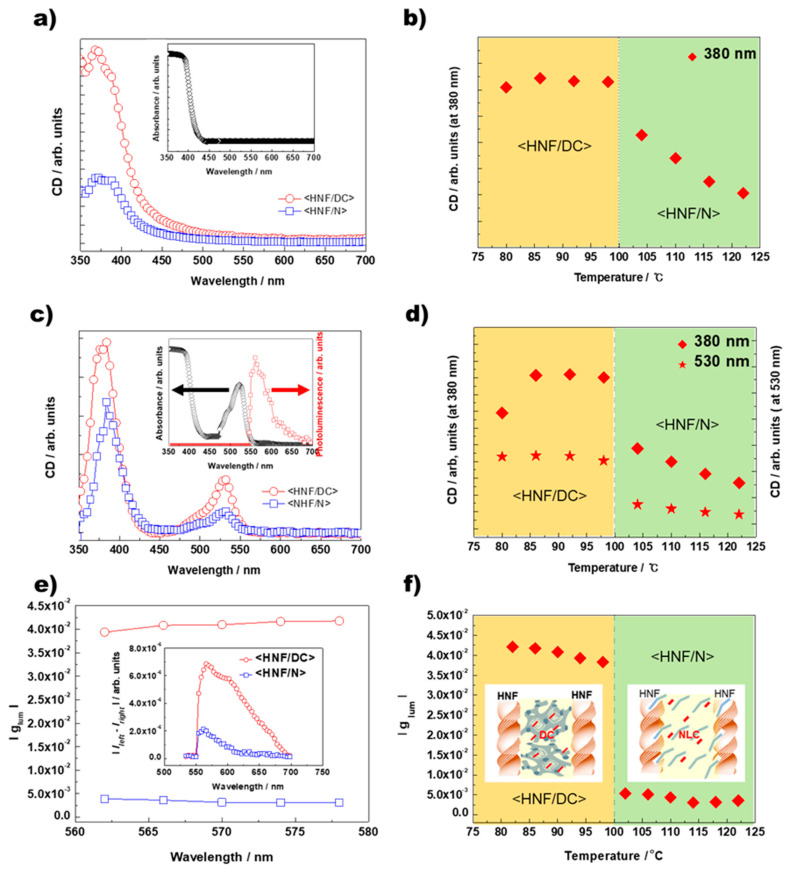
(**a**) Typical CD spectra generated for M1 <HNF/N> and <HNF/DC> positive chiral domains. The M1 absorbance spectrum is also presented in the inset. (**b**) Positive domain maximum CD peak intensity at ~380 nm plotted as a function of temperature. (**c**) Typical CD spectra generated for M2 <HNF/N> and <HNF/DC>. (**d**) Maximum CD peak intensities at ~530 and ~380 nm (originating from dye and BC-1/BC-2 absorbance bands, respectively) plotted as functions of the temperature. (**e**) |g_lum_| evaluated from CPL spectra generated at the <HNF/N> and <HNF/DC> emission bands. Typical <HNF/N> and <HNF/DC> |*I_left_* − *I_right_*| spectra are also shown in the inset. (**f**) |g_lum_| plotted as a function of the temperature. Plausible molecular organizations are also shown for dye molecules (indicated by red bars) embedded in a mixture consisting of two BC molecules at two nanosegregated phases.

Figure 5a shows the typical CD spectra generated for the <HNF/N> and <HNF/DC> positive chiral domains and the M1 absorbance spectrum. The induced CD signals below 450 nm originated from M1 absorbance bands. Figure 5b shows the maximum CD peak intensity of the positive domain at ~380 nm collected for the temperature ranges of <HNF/N> and <HNF/DC>. Fortunately, because the one enantiomeric domain grew to a few millimeters via the mixing of the two BC molecules, we could select the domain showing the positive CD sign at ~380 nm. Interestingly, the peak intensity discretely changed at the phase-transition temperature between <HNF/N> and <HNF/DC>. The <HNF/N> CD intensity originated from the formation of a chiral superstructure composed of NLC (BC-2) embedded in the chiral nanospaces between the BC-1 HNFs [12,14]. At <HNF/DC>, the CD intensity reached the maximum. This suggests that the helicity of the HNF surprisingly affects the chirality of the DC similar to in the N phase. Thus, it is speculated that the chirality of the HNF is transferred to the tilt of the smectic layer in the DC. In the crystal phase below 80 °C, the enhanced birefringence prevents the CD from being precisely evaluated.

A commercial luminescent dye (PM 580) was blended with BC-2. Because the dye trace blended with the BC-2 Iso and N phases negligibly affected the BC-2 molecular ordering and phase sequences, the dye was co-assembled with the BC-2 hierarchical molecular organization. The molecular structure of the luminescent dye is shown in Figure 2a. Figure 5c shows the typical CD spectra generated for <HNF/N> and <HNF/DC> in M2 consisting of BC-1 blended with 40 wt.% of the dye-doped BC-2 (see Table 1). The typical M2 absorbance and photoluminescence spectra are shown in Figure 5c (inset). Both <HNF/N> and <HNF/DC> clearly showed CD signals originating from the dye absorbance band at ~530 nm. Figure 5d shows the maximum CD peak intensities at ~530 and ~380 nm (originating from the dye and BC-1/BC-2 absorbance bands, respectively) plotted for the temperature ranges of <HNF/N> and <HNF/DC>. The M2- and M1-temperature-dependent CD signals (at 380 nm) exhibited almost similar trends, indicating that the dye negligibly affected the BC molecular organization. Moreover, the temperature-dependent CD intensities showed similar trends at 380 and 580 nm, indicating that the induced CDs originating from the dye and both BC molecules are closely related. Notably, the induced CD originating from the dye molecules was influenced by the hierarchically molecularly organized superstructure of the BC-2 molecules embedded in the HNF-generated nanospaces of BC-1.

Figure 5e presents |g_lum_|, as evaluated based on the <HNF/N> and <HNF/DC> CPL spectral emission bands. The |g_lum_| estimates the CPL levels and is defined as |2 (*I_left_* − *I_right_*)/(*I_left_* + *I_right_*)|, where *I_left_* and *I_right_* are the left and right circularly polarized emission magnitudes, respectively [29]. The typical |*I*_left_ − *I*_right_| spectra of <HNF/N> and <HNF/DC> are also displayed in Figure 5e (inset). Figure 5f shows |g_lum_| plotted as a function of the temperature. Similar to the CD trends, the |g_lum_| intensity changes significantly in the vicinity of the phase-transition temperature through <HNF/N> to <HNF/DC>. In <HNF/N>, we observe |g_lum_| in the range of 3–5 × 10^−3^, which is comparable to the previously reported CPL values in another <HNF/N> system [12]. Interestingly, <HNF/DC> reaches a maximum |g_lum_| of ~4.2 × 10^−2^, which is one order of magnitude higher than that of its <HNF/N> counterpart.

Because the dye molecule is inherently achiral, the CD and CPL signals generated at the dye absorption and emission bands, respectively, are attributed to a dye-molecule-organized chiral superstructure. Thus, it is suggested that the different chiroptical performances are attributed to different dye-molecule-organized chiral superstructures in different nanosegregated phases, such as <HNF/N> and <HNF/DC>. Based on the CD and CPL spectra, plausible molecular organizations of the dye molecules embedded in the mixture consisting of two BC molecules at the two nanosegregated phases are illustrated in Figure 5f. In <HNF/N>, the BC-2 N phase segregated from the BC-1 HNFs generated a chiral superstructure in the inter-HNF nanospaces. Hence, meaningful CPL signals are detected from the dye molecules embedded in the N phase co-assembled with the chiral NLC superstructure, as reported by Kim [12]. In <HNF/DC>, the dye molecules are co-assembled with the BC-2 in the tilted smectic layers, and the helicity of the BC-1 HNF may affect the saddle splay deformation of a tilted chiral smectic of the BC-2 DC. In addition, the layer continuity between adjacent layers may become an effective means of propagating layer chirality in the DC phases [11]. As a result, DC exhibits optical rotatory powers comparable to the highest ever found for isotropic fluids of chiral molecules [11]. In this case, the luminescence from co-assembled dye molecules in the DC would be affected by the optical activity of the DC phase, resulting in a strong CPL. Unfortunately, <HNF/DC>, where |g_lum_| reaches a maximum value, is observed in a high-temperature range of 80–100 °C in our case. Thus, we have been trying to lower the temperature range of <HNF/DC> platform for practical applications. Details will be reported in a separate paper soon.

## 3. Materials and Methods

BC-1 and BC-2 were synthesized using the method reported by Akutakawa [30] and Niori [24], respectively. The characteristics of the obtained materials, such as thermotropic polymorphism and transition temperatures, were similar to those reported previously. A luminescent dye molecule (PM580) exhibiting an emission peak in the range of 560–580 nm was purchased from Exciton. The M1 and M2 mixture fractions are summarized in Table 1.

### 3.1. WAXD and RSoXS Analyses

While the samples were cooling, their crystallinities were analyzed using a powder XRD machine (NANOPIX 3.5, Rigaku, Tokyo, Japan) with a Cu-Kα radiation source (*λ* = 0.1542 nm), a camera length of 63.946 mm, and an exposure of time of 10 min per frame (for two frames). The samples were held in a diameter of 1.5 mm glass capillary tube (WJM-Glas/Müller GmbH, Germany) and measured at a constant temperature, which was controlled using a Peltier temperature controller (Rigaku, Tokyo, Japan). The samples were cooled at 5 K min^−1^. The scattering vector *q* (=4πsin*θ λ*^−1^, where 2*θ* = scattering angle) and the detector incident X-ray beam position were calibrated using diffraction orders for several silver behenate crystal layers. The acquired 2D diffraction images were integrated using 2DP software (Rigaku Tokyo, Japan) along the Debye–Scherrer ring to generate the corresponding unidimensional profiles.

Carbon K-edge RSoXS was performed at the BL3U of UVSOR, Institute for Molecular Science, Japan. The X-ray photon energy was adjusted to 284.5 eV, and small-angle scattering (2*θ* ≈ 1°–15°) from the sample was detected with a cooled CCD camera (Newton, Andor, Tokyo, Japan). The samples were sandwiched between 100 nm SiN membrane films and placed in a vacuum chamber. A schematic of the 2D RSoXS experiment is presented in Figure S2.

### 3.2. CD and CPL

The quartz cells used for the CD and CPL measurements were 2 µm thick. A custom setup was built for conducting the CD measurements as shown in Figure S3 (Supplementary Information). White light from a laser-driven light source (EQ-99x, Energetiq Technology, Wilmington, MA, USA) was converted into monochromatic light using a monochromator (Monora 200, DongWoo Optron Co., Ltd., Gwangju, Republic of Korea). A Rochon prism (RPM10, Thorlabs, Newton, NJ, USA) was used to split the beam into two polarization states, of which an ordinary ray passed through a photoelastic modulator (PEM; PEM-100 II/FS47A, Hinds Instruments, Hillsboro, OR, USA). The PEM provided left-handed and right-handed circularly polarized light alternating at a frequency of 47 kHz. The optical power of the light passing through the sample through a 0.5 mm-diameter aperture was detected using a photomultiplier tube (PMT; R928, Hamamatsu Photonics, Shizuoka, Japan) connected to a current amplifier (C11184, Hamamatsu Photonics). The AC component of the output signal (Δ*I*) was measured with a lock-in amplifier (SR830, Stanford Research System, Sunnyvale, CA, USA), while the DC component (*I*) was obtained using a digital multimeter (34410A, Agilent Technologies, Santa Clara, CA, USA). To eliminate artifacts in the CD spectra of the anisotropic solid-state samples originating from linear dichroism and linear birefringence [31], we followed a method proposed by Hirschmann et al., where an artifact-free CD spectrum is determined by averaging the CD spectra for four different sample orientations obtained using 90° in-plane and 180° out-of-plane rotations [32]. For each orientation, five CD measurements were averaged at each wavelength.

A custom setup was built for conducting the CPL measurements as shown in Figure S4 (Supplementary Information). The polarization of the photoluminescence emitted from the sample was modulated by using the PEM oscillating at a frequency of 47 kHz. A linear polarizer was oriented at 45° to the PEM axis. The optical signal was captured using the PMT connected to the side exit port of a spectrometer (SR303i, Andor Technology, Belfast, UK) and amplified using a current amplifier. The DC (*I*) and AC (Δ*I*) components of the signal were measured using a digital multimeter and a lock-in amplifier, respectively. A green LED with a peak wavelength of 530 nm (XP-E, Cree, Inc., Durham, NC, USA) was used to excite the samples through an aperture with a diameter of 1 mm. Artifacts in the CPL spectra due to optical anisotropies originating from the sample and optical components in the setup were eliminated by applying a method proposed by Harada et al. [33]. CPL signals were recorded at different in-plane sample rotations ranging from 0° to 180° in 5° increments, and the maximum and minimum signals were averaged to obtain an artifact-free CPL data.

## 4. Conclusions

Herein, for the first time, we reported the chiroptical performance of nanosegregated <HNF/DC> and compared it with that of <HNF/N>. By blending a guest, BC-2, transiting to the DC through the N phase with a host, BC-1, we obtained two nanosegregated phases, which were identified using POM, WAXD, and RSoXS. Interestingly, enhanced chiroptical performances, such as CD and CPL, at <HNF/DC> were observed and compared with those at <HNF/N>. The maximum |g_lum_| of <HNF/DC> was one order of magnitude higher than that of its <HNF/N> counterpart. The helicity of the BC-1 HNF affected the DC confined between the HNFs, forming an HNF-chirality-associated chiral superstructure, such as the saddle splay deformation of a tilted chiral smectic of the BC-2 DC. It was noted that this chiral superstructure in the sponge-like DC phase without morphological chirality gave rise to enhanced chiroptical performances. The nanosegregated <HNF/DC> phase may provide a useful platform for fabricating practical chiroptical materials without requiring any complex covalent synthesis methods.

## Figures and Tables

**Table 1:** M1 and M2 mixture fractions (wt.%).

	BC-1	BC-2	Dye
M1	60	40	
M2	60	39.2	0.8

## Data Availability

The data supporting the findings of this study are available from the corresponding author upon reasonable request.

## Supplementary Materials

The supporting information can be downloaded at: https://www.mdpi.com/article/10.3390/ijms232314629/s1.

## Abbreviations

LC, liquid crystal; BC, bent core; HNF, helical nanofilament; DC, dark conglomerate; CPL, circularly polarized luminescence; NLC, nematic liquid crystal; CD, circular dichroism; POM, polarized optical microscopy; XRD, X-ray diffraction; Iso, isotropic; g_lum_, luminescence dissymmetry factor; RSoXS, resonant soft X-ray scattering; WAXD, wide-angle X-ray diffraction; DSC, differential scanning calorimetry; PEM, photoelastic modulator.
