# Supplementary material for: Chiroptical Performances in Self-Assembled Hierarchical Nanosegregated Chiral Intermediate Phases Composed of Two Different Achiral Bent-Core Molecules
Source: Int J Mol Sci. 2022 Nov 23;23(23):14629. doi: 10.3390/ijms232314629 (PMC9736540; doi:10.3390/ijms232314629)
Supplement: Supplementary file 1 [file ijms-23-14629-s001.zip › ijms-2005598-supplementary.pdf]

Supporting Information

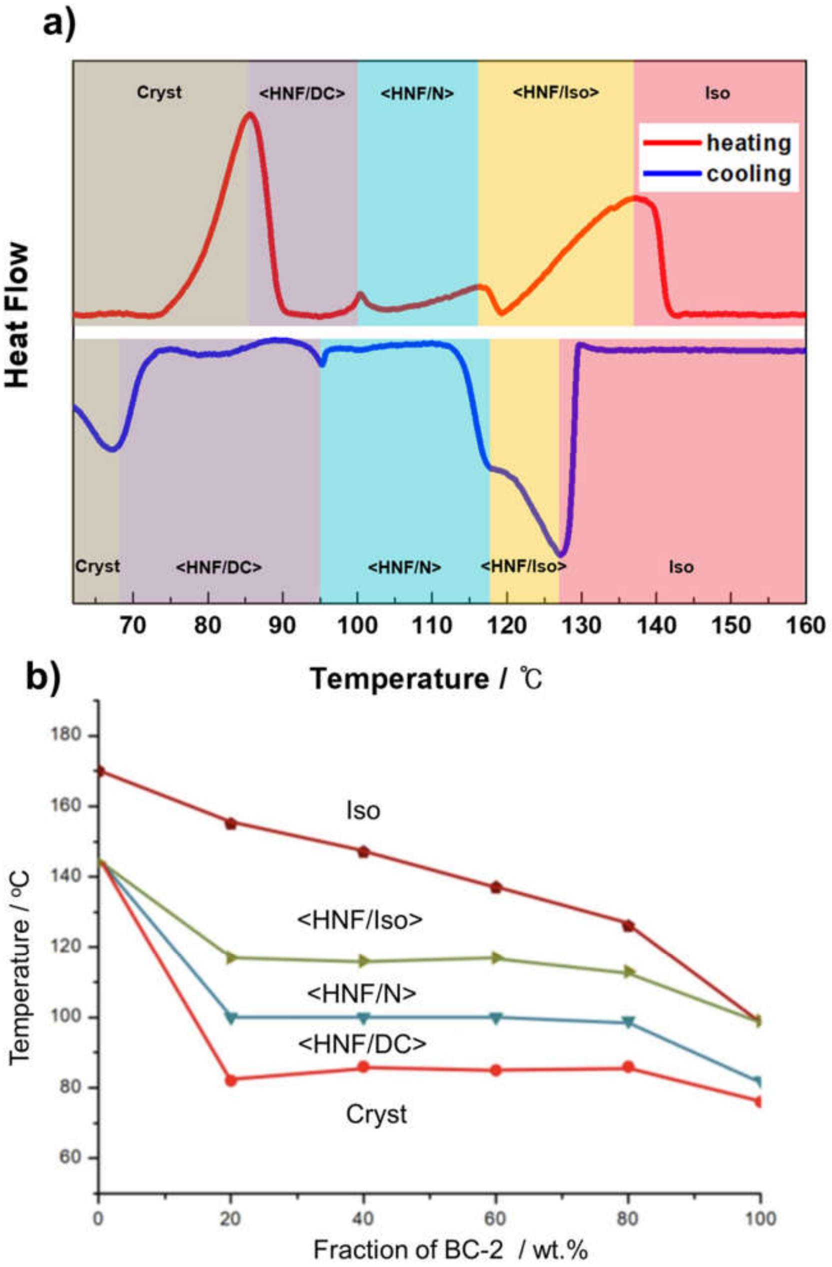

Figure S1. (a) DSC profile of the mixture composed of BC-1 (host) and 40 wt.% BC-2 (guest). (b) Phase diagram of binary mixture systems with different fractions.

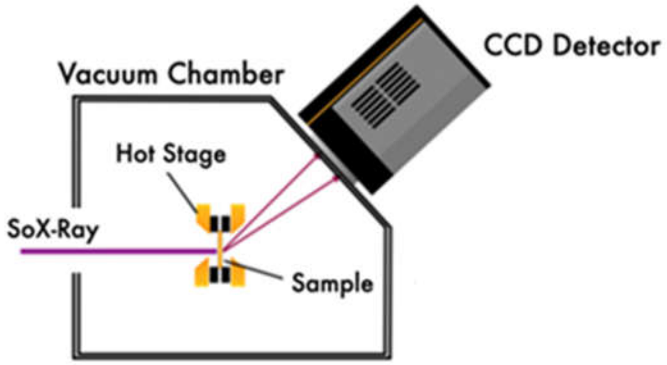

Figure S2. Schematic of the two-dimensional resonant soft X-ray scattering experiment.

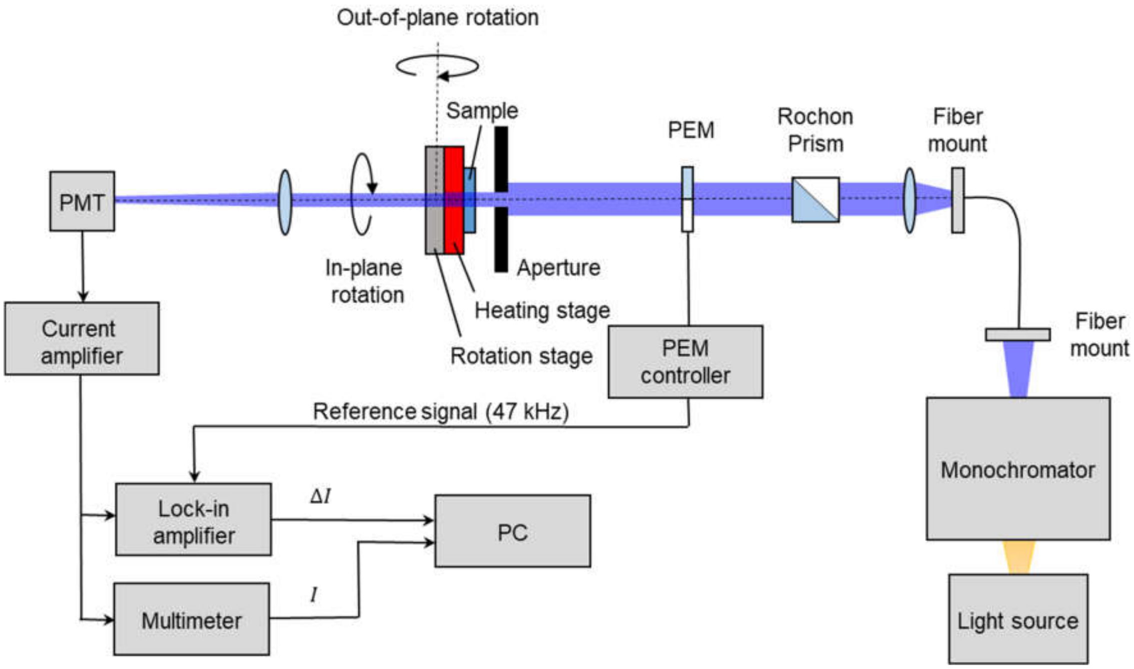

Figure S3. Schematic of the custom-made circular dichroism spectroscopic system.

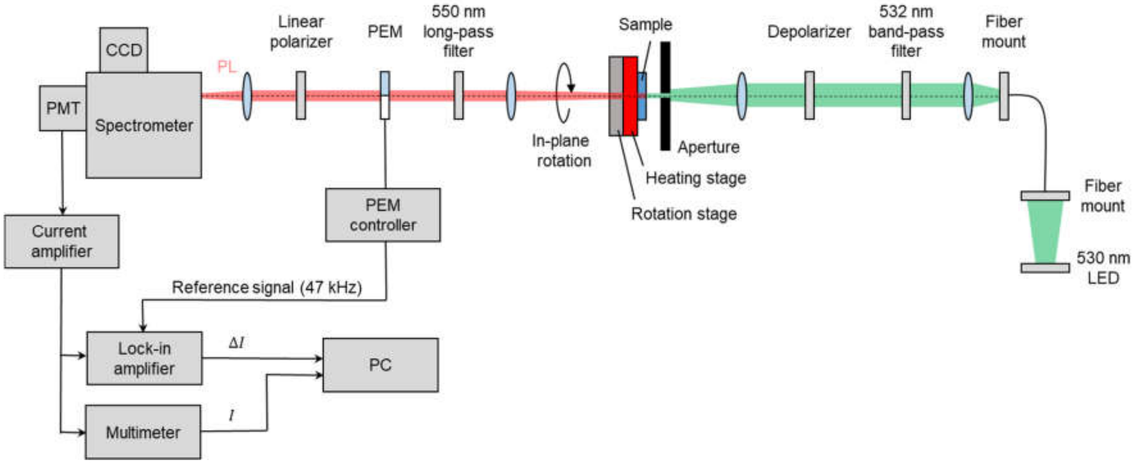

Figure S4. Schematic of the custom-made circularly polarized luminescence (CPL) spectroscopic system.
